# The Impact of Previous Comorbidities on New Comorbidities and Medications after a Mild SARS-CoV-2 Infection in a Lithuanian Cohort

**DOI:** 10.3390/jcm13020623

**Published:** 2024-01-22

**Authors:** Dovilė Važgėlienė, Raimondas Kubilius, Indre Bileviciute-Ljungar

**Affiliations:** 1 Department of Physical Rehabilitation Medicine, Kaunas Clinic of the Lithuanian University of Health Sciences, 50161 Kaunas, Lithuaniaraimondas.kubilius@lsmuni.lt (R.K.); 2 Department of Nursing, Lithuanian University of Health Sciences, 44307 Kaunas, Lithuania; 3 Department of Clinical Sciences, Karolinska Institute, Danderyd University Hospital, 182 57 Stockholm, Sweden; 4 Multidisciplinary Pain Clinic, Capio St. Göran Hospital, 112 19 Stockholm, Sweden

**Keywords:** SARS-CoV-2 virus, medication, comorbidities, post-COVID-19 condition, vitamins/supplements

## Abstract

This cross-sectional study investigates new comorbidities and new medications after a mild SARS-CoV-2 infection. Data were collected after an acute SARS-CoV-2 infection by online survey in a Lithuanian cohort. Sociodemographic data, SARS-CoV-2-related symptoms, previous and new comorbidities, and medications were analysed. The results of 895 participants (mean age: 44 years) show that 91% were women, 58% had higher education, and 84% were working. Among those, 473 (52.8%) answered being “healthy” before infection; 823 (92%) indicated being positive on diagnostic tests; and 841 (94%) were non-hospitalized. Asymptomatic infection was reported by 17 participants (1.9%). Participants reporting any comorbidity before a SARS-CoV-2 infection reported more frequently having remaining symptoms compared to those who were “healthy”, particularly in relation to neurological symptoms. Thirteen percent of participants reported new comorbidities, and thirty-five percent started new medication. Among new medications, an intake of vitamins/supplements (21%) and anti-inflammatory drugs (4%) was more often reported by “unhealthy” participants. Regression analysis revealed that new cardiovascular and pulmonary diagnoses predicted each other. Participants reporting prior neurological disorders tended to have an increased risk of intaking new vitamins/supplements and anti-inflammatory drugs after infection. The results indicate a significantly increased consumption of medication, particularly unprescribed substances, after SARS-CoV-2, indicating a need of more research in this area.

## 1. Introduction

Ailments that linger longer than three months after a SARS-CoV-2 infection and affect daily life activities are known as a post-COVID-19 condition, according to World Health Organization (WHO) [1]. According to WHO, approximately 10% of all infected people may suffer from a post-COVID-19 condition [1]. However, the epidemiological data are not clear. In Sweden, a study analysing data from the health care system for the period 2021–2022 found that approximately 2% of population of 4.1 million have been diagnosed with a post-COVID-19 condition after an acute SARS-CoV-2 infection [2] Among them, the majority were non-hospitalised inhabitants.

Theoretically, previous comorbidities might increase the severity of a post-COVID-19 condition, although studies examining this particular risk factor are few, especially after a mild SARS-CoV-2 infection. In 2021, Kayaaslan et al. have already reported that persistent symptoms in both inpatients and outpatients after SARS-CoV-2 were predicted by previous comorbidities [3]; however, they were not confirmed by others [4]. In a large cohort including mainly females and health care workers, previous mental distress was reported to be a risk factor for those with a post-COVID-19 condition [5]. Moreover, a recent systemic review and meta-analysis revealed several risk factors, i.e., demographic (age, sex), higher body mass index, smoking, and comorbidities such as depression, cardiovascular, pulmonary, endocrine, and immune suppression diseases, while vaccination lowered the risk by approximately at 40% [6]. Recently, we reported data from the first survey (first pandemic wave) of a Lithuanian cohort showing that both previous comorbidities and medication increased symptomatology during an acute infection and after 28 days [7].

The aim of this study was to study further previous and new comorbidities as well as previous and new medications after a SARS-CoV-2 infection in mainly non-hospitalized persons in a Lithuanian cohort during the pandemic’s second wave in 2021–2022. The hypothesis was that participants with previous comorbidities will report more remaining symptoms and more new comorbidities and medication use.

## 2. Materials and Methods

This study was performed by inviting participants to answer anonymously an Internet-based questionnaire, created by D.V. via Google Drive (Alphabet Inc., Googleplex, Mountain View, CA, USA). The questionnaire was distributed in the Lithuanian language through Lithuanian websites, including private/public Facebook groups, city/town/district hospitals, and media outlets (Supplementary Material S1). Study encouraged participation independent of the presence or absence of persistent symptoms. Ethical approval was obtained from the Kaunas Regional Ethics Committee for Biomedical Research on the 11th of May 2021 (approval number: BE-2-65). This study has been registered with ClinicalTrials.gov (ID: NCT05000229). Informed consent was obtained from each participant. The study protocol, materials concerning ethical permission, and consent information provided to participants are available at the university’s website (https://lsmu.lt/en/about-lsmu/structure/medical-academy/faculty-of-nursing/projektine-veikla/, accessed on 22 December 2021). Questions were formulated to gather information regarding sociodemographic characteristics, the data of acute SARS-CoV-2 infection, including diagnostics tests, information related to comorbidities, and the daily use of medication before and after an infection, remaining symptoms after the infection, and attitudes regarding the need for rehabilitation. The questions were of exploratory nature with free or predefined answers. Here, we present a second collection of the survey (from 10 August 2021 to 31 December 2022), excluding questions related to the need for rehabilitation.

Inclusion criteria for participation were as follows: (1) age of 18 years or older, (2) known SARS-CoV-2 infection with or without specific diagnostic tests (PCR, antigen test, antibodies), and (3) a post-infection period of at least 28 days before participating in the survey.

Exclusion criteria were as follows: hospitalized patients still receiving treatment or rehabilitation after a SARS-CoV-2 infection, unstable or untreated comorbidities, or the ongoing stabilisation of comorbidities. None of the participants indicated the presence of exclusion criteria in the questionnaire.

For persistent symptoms, we created 64 preselected answers as well as the possibility to leave free comments regarding other symptoms. Persistent symptoms were formed into major groups as indicated in Supplementary Material S2.

For comorbidities, the participants were asked to answer the presence of any chronic disease prior to and after a SARS-CoV-2 infection. In cases of previous and recent disease, the participant was asked to specify the ailment. Furthermore, the comorbidities could be selected from 21 preselected disorders as well as the possibility to leave free comments regarding other diseases. For medication, the participants were asked for the presence of any daily medication before and after a SARS-CoV-2 infection. In cases with previous and recent medication, the participant was asked to specify the medication. Both comorbidities and medications were grouped as indicated in the respective Supplements (see Methods).

### Statistics

Results from the survey were analysed with SPSS version 29 (Statistical Package for the Social Sciences, IBM, New York, NY, USA) after downloading into a Microsoft Excel 2019 file (Microsoft Corporation, Washington, DC, USA). A major part of data was nominal in nature, and therefore, presented as a number of participants and as a percentage of the whole cohort. Nominal data were compared using two-tailed Chi-square test. For parametric data (the duration of symptoms), an independent *t*-test was applied between the groups (“healthy” vs. “unhealthy”). The predictors of new comorbidities and new medications were analysed with separate binary logistic regression models for those dependent variables, showing significant differences between the groups and even including sex as an additional covariate. Statistical significance was considered as *p* < 0.05.

## 3. Results

### 3.1. Participants and Persistent Symptoms in Relation to Previous Health Status

The survey of participants is presented in Supplementary Material S1. The final data analysis included 895 participants with a mean age of 44 years (SD: 12.54 years, range: 18–79 years). Nearly 90% of participants were females and younger than 60 years (Table 1). Approximately 65% participants were from the three biggest cities in Lithuania, including Kaunas (the second-largest city in Lithuania, 27.6%), Vilnius (the capital of Lithuania, 26.3%), and Klaipėda (11.8%); therefore 72.0% participants were living in an urban environment (Table 1). Diagnostic testing was reported by 92.0% of participants, and an asymptomatic infection by approximately 2%. Approximately 71% of participants reported being vaccinated, with no difference observed between “healthy” and “unhealthy” participants (Chi-Square test, *p* = 0.47). The median duration after a SARS-CoV-2 infection and the responding survey was 27 weeks (range: 4–129 weeks, mean: 30 weeks, and SD: 22 weeks), with no difference found between “healthy” and “unhealthy” participants (independent *t*-test, *p* = 0.36). Approximately 61% of participants reported to be “officially” healthy from the acute SARS-CoV-2 infection within 2 weeks, 27% within 2–4 weeks, and 10% after 4 weeks.

The majority od participants (94.0%) reported being non-hospitalized, whereas 5% reported being hospitalized during an acute SARS-CoV-2 infection. The results show that “unhealthy” participants were older, among them many were retired and unemployed, see Table 1. No statistical difference was revealed regarding sex for “healthy” and “unhealthy” participants.

Approximately 92.2% of participants reported persistent symptoms with a total number ranging from 0 to 45 (median 7 symptoms). All 64 symptoms are presented in Supplementary Material S2. The most frequently reported remaining symptoms were related to the nervous system and chronic pain, followed by symptoms of the upper respiratory tract, see Table 2. Thereafter, symptoms related to the cardiovascular system, skin, mood/emotions, the lower respiratory tract, the endocrine system, vision, and the gastrointestinal tract were reported. Participants reporting any comorbidity before SARS-CoV-2 infection (“unhealthy”) mentioned all remaining symptoms more frequently compared to those who did not report any symptoms (“healthy”), except for psychological symptoms, Table 2.

### 3.2. Comorbidities and Medications Prior to SARS-CoV-2 Infection

Approximately 53% of participants reported being “healthy” prior to their SARS-CoV-2 infection. Comorbidities were grouped according to major disease groups and presented in Table 3. High blood pressure dominated among cardiovascular diseases (*N* = 172 or 19.2%); obesity among endocrine diseases (*N* = 90 or 10.1%); sleep disorders among neurological diseases (*N* = 62 or 6.9%); anxiety among psychiatric diseases (*N* = 43 or 4.8%); thereafter unspecified diseases of the gastrointestinal tract (*N* = 68 or 7.6%); unspecified allergic diseases (*N* = 49 or 5.5%); unspecified rheumatic diseases (*N* = 49 or 5.5%); asthma among pulmonary diseases (*N* = 37 or 4.1%); unspecified kidney diseases (*N* = 18 or 2%); unspecified oncological diseases (*N* = 17 or 1.9%); unspecified immunodeficiency diseases (*N* = 7 or 0.8%); and others (*N* = 22 or 2.5%) (Table 3).

Of those with comorbidities, the greatest portion had one comorbidity (20.1%), and only two (0.2%) participants reported nine comorbidities. The total number of comorbidities varied from zero to nine, with a median of two comorbidities.

Approximately 68% of participants reported not taking any medication before a SARS-CoV-2 infection. Among those reporting medication, the cardiovascular system-modulating drugs were consumed by 12% of participants, followed by hormones (7%) and “other” drugs (12%). Psychopharmacological, anti-allergic, anti-inflammatory/anti-bacterial, and supplements/vitamins were taken by certain percentages of participants in the whole cohort, see Table 4. As expected, “unhealthy” participants more often reported a daily pharmacological treatment before an acute infection.

### 3.3. New Comorbidities and Medications after SARS-CoV-2 Infection

One hundred and fourteen participants reported new diagnoses. Among them, 82 participants received one new diagnosis, 25 participants received two new diagnoses, 4 participants received three new diagnoses, and 3 participants received four new diagnoses. Cardiovascular, neurological, and pulmonary diagnoses were new diagnoses reported by 3.5%, 2.6%, and 1.8% of participants, respectively, see Table 5. “Other” diagnoses were reported by 3.1% participants, and the remaining diagnoses (gastrointestinal, endocrine, inflammatory/rheumatic, renal, dermatological, psychiatric, and gynaecological) were reported by approximately 1% or less of participants per each diagnosis, see Table 5. A significant difference was found between “healthy” and “unhealthy” participants regarding new cardiovascular and pulmonary diagnoses, which were more often reported by “unhealthy” participants, see Table 2.

Three hundred and thirteen participants started new medications. Among them, 228 participants started one new medication, 74 started two, 10 started three, and 1 started four medications/supplements/vitamins. The results show that supplements and vitamins were most frequently reported as new medications (by almost 21% of participants), followed by “other” drugs (reported by approximately 11% of participants) and cardiovascular drugs (reported by approximately 6% of participants), see Table 6. Less than 5% of participants reported new anti-inflammatory and psychopharmacological drugs, while new endocrine- and/or immune system-regulating drugs were consumed by less than 1% of the cohort. Statistical analysis revealed that “unhealthy” participants more often reported taking supplements and vitamins and anti-inflammatory/antibacterial drugs compared to “healthy” ones, see Table 6.

### 3.4. Regression Analysis for Predictors of New Comorbidities and New Medications after SARS-CoV-2 Infection

The predictors of new comorbidities and new medications were analysed with separate binary logistic regression models for the dependent variables, showing significant differences between the groups. Sex was an additional covariate in the analysis since women were overrepresented in the study cohort. To analyse the predictors for new cardiovascular and pulmonary diagnoses, we chose the following covariates: age group, sex, socioeconomic situation, new daily intake of vitamins/supplements, anti-inflammatory drugs, and all three major prior comorbidities (cardiovascular, endocrine, and neurological). To analyse the predictors for new daily intake of vitamins/supplements and anti-inflammatory drugs, we choose the following covariates: age group, sex, socioeconomic situation, new cardiovascular and pulmonary diagnoses, and all three major prior comorbidities (cardiovascular, endocrine, and neurological). Table 7 summarises the un-adjusted regression coefficients, showing that new cardiovascular diseases were predicted due to new pulmonary diseases and vice versa with odds of up to 5.1–5.2 (95% CI: 1.3–20).

The intake of new vitamins/supplements was slightly predicted by sex with odds of up to 0.33 (95% CI 0.15–0.74). A previous neurological disease also showed a tendency to predict the intake of new vitamins/supplements with odds up of up to 1.6 (95% CI: 1.0–2.6, *p* = 0.059).

We did not find any significant predictors for new anti-inflammatory drugs, except for a prior neurological disease showing a tendency with odds of up to 2.2 (95% CI: 0.9–5.1, *p* = 0.074).

## 4. Discussion

The results of the second survey confirm that neurological symptoms (fatigue, neurocognitive issues, sleep-related symptoms, and pain) were the most reported by participants during the second wave of pandemics. Among comorbidities prior to SARS-CoV-2 infection, cardiovascular, endocrine, and neurological diseases dominated, even among the middle-aged population, mostly employed women. As expected, and as reported previously [7], those with any chronic disease prior to an infection (“unhealthy”) more often reported persistent symptoms and took daily medication. Those categorised as “unhealthy” also more frequently reported new diagnoses and new medications after an infection. Among new diagnoses, cardiovascular issues were the most frequent, having been reported by 3.5% of participants. In the present study population, cardiovascular comorbidities already dominated prior to a SARS-CoV-2 infection, which could naturally result in more frequent new cardiovascular diagnoses after an infection. Alternatively, 6.1% of participants reported new cardiovascular drugs without a statistically significant difference between the “healthy” vs. “unhealthy” participants. Studies regarding an increased risk for acute myocardial infarction and ischaemic stroke following COVID infection have been published [8], whereas postural orthostatic tachycardia syndrome (POTS) as a sign of dysregulation in the autonomic nervous system has been reported as a symptom of the post-COVID-19 condition [9]. The molecular mechanisms behind cardiovascular symptoms in a post-COVID-19 condition are unknown, but recently, the dysregulation of the proteome, cytokines, chemokines, and sphingolipid levels has been reported [10]. Another study reveals a prevalence of low vitamin D among 447 post-COVID-19 patients but did not find any difference in the prevalence of symptoms or symptom severity between low and normal vitamin D groups [11].

Unexpectedly, we found an increase from 1% to almost 21% in the consumption of unprescribed vitamins/supplements after a SARS-CoV-2 infection, which was very slightly influenced by the female sex. Self-medication, including supplements and vitamins, have been studied mostly during an acute COVID infection [12]. Carrasco-Garrido and colleagues reported an increased consumption of psychopharmacological substances in the anonymously collected data of Spanish participants with post-COVID-19 symptoms [13], where almost 45% of 391 participants reported taking benzodiazepines and Z-hypnotics; however, the study did not report if it was a new medication or an already established intake before a SARS-CoV-2 infection. Another study reported an increased burden on the health care system at six months after a SARS-CoV-2 infection, where the prescription of medication(s) was a part of the burden but was not analysed in detail [14]. To our knowledge, there is no study examining the patterns of the consumption of prescribed and unprescribed drugs, including vitamins and supplements, related to a post-COVID-19 condition. Some studies investigated the effects of supplements/vitamins, for example, reporting the positive effects of 1-arginine plus vitamin C supplementation [15] and fermented tropical fruits [16] in randomised controlled trials in participants with a post-COVID-19 condition. However, a recent systematic review of 39 randomised controlled studies on eight dietary supplements protecting the immune system against stressors in healthy individuals did not show conclusive results [17]. Therefore, a careful anamnesis of unprescribed medication should be included in clinical practice for patients with a post-COVID-19 condition, especially for female patients with previous comorbidities related to the nervous system.

The daily intake of new anti-inflammatory substances was reported by 4.2% of participants, more often by “unhealthy” participants. We hypothesize that an increased intake of new medications might be rather predicted by the remaining symptoms than comorbidities and needs further exploration. Taken together, prior neurological disorders were reported by approximately 12% of the study cohort and tended to be associated with an increased intake of vitamins/supplements and anti-inflammatory drugs (non-steroidal anti-inflammatory drugs or antibiotics) after an infection. Both vitamins and supplements, but not antibiotics, could be obtained as over-the-counter medications, which indicates an increased consumption of unprescribed drugs in people with persistent post-infectious symptoms. Therefore, in the next step, we will analyse a consumption of new medications in terms of symptomatology since neurological symptoms were predominant in the study cohort.

This study has several limitations: (1) the generalizability is limited by recruitment through social media and the representation of mainly middle-aged women; (2) the limited control of gathered data due to anonymity and self-reported data; (3) the absence of information if medication was prescribed or obtained over-the-counter and the reason for the prescription; and (4) the retrospective collection of data regarding previous medications and comorbidities. The present study was started prior to WHO defining a post-COVID-19 condition [1]. Therefore, it reports rather on the health status of participants after a SARS-CoV-2 infection than on a post-COVID-19 condition.

Following are the strengths of the study: (1) the questionnaire covered a broad spectrum of comorbidities and medications both before and after an infection and (2) the expanded analysis of comorbidities and medications was performed by three independent clinicians.

## 5. Conclusions

In conclusion, the results of this study revealed that previous comorbidities are associated with more persistent symptoms, increased new comorbidities, and new prescribed and unprescribed medications after a SARS-CoV-2 infection. Particularly, unprescribed vitamins/supplements and anti-inflammatory drugs should be inquired about during the clinical evaluations of patients with a post-COVID-19 condition, especially when involving female patients. Since the clinical value of unprescribed medications used for a post-COVID-19 condition is not yet known, more research in this area is warranted.

## Figures and Tables

**Table 1:** The socioeconomic data of participants presented as numbers and percentages of a whole cohort and among those reported as “healthy” and “unhealthy” prior to the infection.

		All *N* = 895 (100%)	“Healthy” *N* = 473 (52.8%)	“Unhealthy” *N* = 422 (47.2%)	Statistics
Sex	Female	816 (91.2%)	431 (48.2%)	385 (43.0%)	0.524
	Male	79 (8.8%)	42 (4.7%)	37 (4.1%)	
Age group	Younger than 40 years	373 (41.7%)	269 (30.1%)	104 (11.6%)	<0.001
	41–60 years	418 (46.7%)	187 (20.9%)	231 (25.8%)	
	61–80years	104 (11.6%)	17 (1.9%)	87 (9.7%)	
Education	Primary/secondary	106 (11.8%)	49 (5.5%)	57 (6.4%)	0.066
	Higher non-university	266 (29.7%)	129 (14.4%)	137 (15.53%)	
	Higher university	520 (58.1%)	294 (32.8%)	226 (25.3%)	
	Other	3 (0.3%)	1 (0.1%)	2 (0.2%)	
Socioeconomic situation	Employed/working	749 (83.7%)	426 (47.6%)	323 (36.1%)	<0.001
	Temporary unemployed	30 (3.4%)	10 (1.1%)	20 (2.2%)	
	Unemployed	55 (6.0%)	26 (2.9%)	29 (3.2%)	
	Retired	52 (5.8%)	7 (0.8%)	45 (5.0%)	
	Student	9 (1.0%)	5 (0.6%)	4 (0.4%)	
Region of Residence in Lithuania	Kaunas	247 (27.6%)	140 (15.6%)	107 (12.0%)	0.359
	Vilnius	236 (26.3%)	126 (14.1%)	110 (12.3%)	
	Klaipėda	106 (11.8%)	52 (5.8%)	54 (6.0%)	
	Šiauliai	75 (8.3%)	42 (4.7%)	33 (3.7%)	
	Panevėžys	73 (8.2%)	33 (3.7%)	40 (4.5%)	
	Telšiai	40 (4.5%)	22 (2.5%)	18 (2.0%)	
	Marijampolė	36 (4.0%)	16 (1.8%)	20 (2.2%)	
	Alytus	33 (3.7%)	16 (1.8%)	17 (1.9%)	
	Utena	31 (3.5%)	20 (2.2%)	11 (1.2%)	
	Tauragė	18 (2.0%)	6 (0.7%)	12 (1.3%)	
Living area	Settlement	41 (4.6%)	17 (1.9%)	24 (2.7%)	0.224
	Village	101 (11.3%)	51 (5.7%)	50 (5.6%)	
	City	644 (72.0%)	340 (38.0%)	304 (34.0%)	
	Suburbs	109 (12.2%)	65 (7.3%)	44 (4.9%)	

**Table 2:** Groups according to remaining symptoms presented as the number and percentage of a whole cohort and among those reported as “healthy” and “unhealthy” prior to the infection. Differences between these two latter groups are presented as *p*-values. Originally marked symptoms are presented in Supplementary Material S2.

Symptoms Related to	All *N* = 895 (100%)	“Healthy” *N* = 473 (52.8%)	“Unhealthy” *N* = 422 (47.2%)	Statistics
Nervous system	739 (82.6%)	370 (41.3%)	369 (42.2%)	*p* < 0.001
Chronic pain	477 (53.3%)	219 (24.5%)	258 (28.8%)	*p* < 0.001
Throat, nose, and ear	420 (46.9%)	203 (22.7%)	217 (24.2.6%)	*p* = 0.007
Heart	364 (40.7%)	156 (17.4%)	208 (23.2%)	*p* < 0.001
Skin	335 (37.4%)	149 (16.6%)	186 (20.8%)	*p* < 0.001
Mood and emotions	307 (34.3)	165 (18.4%)	142 (15.9%)	*p* = 0.375
Lung	258 (28.8%)	116 (13.0%)	142 (15.9%)	*p* = 0.002
Endocrine	254 (28.4%)	117 (13.1%)	137 (15.3%)	*p* = 0.006
Vision and eyes	246 (27.5%)	103 (11.5%)	143 (16.0%)	*p* < 0.001
Gastrointestinal tract	165 (18.4%)	73 (8.2%)	92 (10.3%)	*p* = 0.009
Other	601 (67.2%)	291 (32.5%)	310 (34.6%)	*p* < 0.001

**Table 3:** Grouped comorbidities in “unhealthy” participants are presented as a number and a percentage of a whole cohort. Originally marked comorbidities and groupings are presented in Supplementary Material S3.

Disorders	All *N* = 895 (100%)	“Healthy” *N* = 473 (52.8%)	“Unhealthy” *N* = 422 (47.2%)	Statistics
Cardiovascular	209 (23.4%)	0	209 (23.4%)	*p* < 0.001
Endocrine	158 (17.7%)	0	158 (17.7%)	*p* < 0.001
Neurological	111 (12.4%)	0	111 (12.4%)	*p* < 0.001
Gastrointestinal	68 (7.6%)	0	68 (7.6%)	*p* < 0.001
Psychiatric	63 (7.0%)	0	63 (7.0%)	*p* < 0.001
Skin	49 (5.5%)	0	49 (5.5%)	*p* < 0.001
Inflammatory rheumatic	49 (5.5%)	0	49 (5.5%)	*p* < 0.001
Pulmonary	49 (5.5%)	0	49 (5.5%)	*p* < 0.001
Renal	18 (2.0%)	0	18 (2.0%)	*p* < 0.001
Oncological	17 (1.9%)	0	17 (1.9%)	*p* < 0.001
Immunodeficiency	7 (0.8%)	0	7 (0.8%)	*p* = 0.005
Others	22 (2.5%)	0	22 (2.5%)	*p* < 0.001

**Table 4:** Grouped medications before a SARS-CoV-2 infection presented as a number and a percentage in a whole cohort and among those reported as “healthy” and “unhealthy” prior to the infection. Differences between these two latter groups are presented as *p*-values. Originally marked medications and groupings are presented in Supplementary Material S4.

Drug Regulating	All *N* = 895 (100%)	“Healthy” *N* = 473 (52.8%)	“Unhealthy” *N* = 422 (47.2%)	Statistics
The cardiovascular system	109 (12.2%)	11 (2.3%)	98 (10.9%)	*p* < 0.001
The endocrine system	61 (6.8%)	10 (1.1%)	51 (5.7%)	*p* < 0.001
Psychological functions (psychopharmacology)	23 (2.6%)	0 (0.0)	23 (2.6%)	*p* < 0.001
Inflammation (nonsteroidal anti-inflammatory drugs and antibiotics)	13 (1.5%)	2 (0.2%)	11 (1.2%)	*p* = 0.006
The immune system (antiallergic and anti-asthmatic)	10 (1.1%)	0 (0.0)	10 (1.1%)	*p* < 0.001
Gastrointestinal tract	10 (1.1%)	0 (0.0)	10 (1.1%)	*p* < 0.001
Supplements/vitamins	9 (1.0%)	2 (0.2%)	7 (0.8%)	*p* = 0.064
Other	105 (11.7%)	9 (1.0%)	96 (10.7%)	*p* < 0.001

**Table 5:** New diagnoses after a SARS-CoV-2 infection are presented as a number and a percentage in a whole cohort and among those reported as “healthy” and “unhealthy” prior to the infection. Differences between these two latter groups are presented as *p*-values. Originally marked comorbidities and groupings are presented in Supplementary Material S3.

	All *N* = 895 (100%)	“Healthy” *N* = 473 (52.8%)	“Unhealthy” *N* = 422 (47.2%)	Statistics
Cardiovascular	31 (3.5%)	9 (1.0%)	22 (2.5%)	*p* = 0.006
Neurological	23 (2.6%)	9 (1.0%)	14 (1.6%)	*p* = 0.131
Pulmonary	16 (1.8%)	4 (0.4%)	12 (1.3%)	*p* = 0.022
Gastrointestinal	12 (1.3%)	6 (0.7%)	6 (0.7%)	*p* = 0.534
Endocrine	11 (1.2%)	3 (0.3%)	8 (0.9%)	*p* = 0.079
Inflammatory rheumatic	8 (0.9%)	2 (0.2%)	6 (0.7%)	*p* = 0.109
Renal	6 (0.7%)	1 (0.1%)	5 (0.6%)	*p* = 0.084
Dermatological	6 (0.7%)	3 (0.3%)	3 (0.3%)	*p* = 0.602
Psychiatric	5 (0.6%)	2 (0.2%)	3 (0.3%)	*p* = 0.447
Gynaecological	1 (0.1%)	1 (0.1%)	0 (0%)	*p* = 0.528
Others	28 (3.1%)	13 (1.5%)	15 (1.7%)	*p* = 0.308

**Table 6:** New medications after a SARS-CoV-2 infection presented as a number and a percentage in a whole cohort and among those reported as “healthy” and “unhealthy” prior to the infection. Differences between these two latter groups are presented as *p*-values. Originally marked medications and groupings are presented in Supplementary Material S4.

Drugs Regulating:	All *N* = 895 (100%)	“Healthy” *N* = 473 (52.8%)	“Unhealthy” *N* = 422 (47.2%)	Statistics
Supplements/vitamins	187 (20.9%)	82 (9.2%)	105 (11.7%)	*p* = 0.004
The cardiovascular system	55 (6.1%)	26 (2.9%)	29 (3.2%)	*p* = 0.237
Inflammation (nonsteroidal anti-inflammatory drugs and antibiotics)	38 (4.2%)	11 (1.2%)	27 (3.0%)	*p* = 0.002
Psychological functions (psychopharmacology)	22 (2.5%)	8 (0.9%)	14 (1.6%)	*p* = 0.127
The immune system (antiallergic and anti-asthmatic)	6 (0.7%)	2 (0.2%)	4 (0.4%)	*p* = 0.291
The endocrine system	8 (0.9%)	2 (0.4%)	6 (0.5%)	*p* = 0.151
Other	96 (10.7%)	43 (4.8%)	53 (5.9%)	*p* = 0.059

**Table 7:** Regressors predicting new diagnoses and medication after a SARS-CoV-2 infection.

Regressors	New Cardiovascular Disease OR (95% CI), *p*-Value	New Pulmonary Disease OR (95% CI), *p*-Value	New Vitamins/Supplements OR (95% CI), *p*-Value	New Anti-Inflammatory Drugs OR (95% CI), *p*-Value
Age group	n.s.	n.s.	n.s.	n.s.
Sex	n.s.	n.s.	0.33 (0.15–0.74), *p* = 0.007	n.s.
Sociodemographic characteristics	n.s.	n.s.	n.s.	n.s.
Prior cardiovascular diseases	n.s.	n.s.	n.s.	n.s.
Prior endocrine diseases	n.s.	n.s.	n.s.	n.s.
Prior nervous system diseases	n.s.	n.s.	n.s.	n.s.
New cardiovascular disease	-	5.24 (1.33–20.55), 0.018	n.s.	n.s.
New pulmonary disease	5.1 (1.3–19.76), 0.02	-	n.s.	n.s.
New vitamins/supplements	n.s.	n.s.	-	n.s.
New anti-inflammatory drugs	n.s.	n.s.	n.s.	-

Abbreviations: n.s. = not significant.

## Data Availability

The data that support the findings of this study are available from the first author (D.V.) upon reasonable request.

## Supplementary Materials

The following supporting information can be downloaded at: https://www.mdpi.com/article/10.3390/jcm13020623/s1, Supplementary Materials S1 includes links to websites for study dissemination and flow figure of survey. Supplementary Materials S2 includes all persistent symtoms of the study cohort and grouping of symptoms. Supplementary Materials S3 includes grouping of comorbidities and Supplementary Materials S4 includes grouping of medication.
