# Supplementary material for: The Impact of Previous Comorbidities on New Comorbidities and Medications after a Mild SARS-CoV-2 Infection in a Lithuanian Cohort
Source: J Clin Med. 2024 Jan 22;13(2):623. doi: 10.3390/jcm13020623 (PMC10816360; doi:10.3390/jcm13020623)
Supplement: Supplementary file 1 [file jcm-13-00623-s001.zip › jcm-2818971-supplementary.pdf]

Supplemental Material S1.
Links to websites where the information about the study was distributed.

https://www.kaunoklinikos.lt/apie-mus/naujienos/covid-19-liga-kokie-pozymiai-ir-liekamieji-reiskiniai-labiausiai-vargina-lietuvius.html
https://www.facebook.com/groups/Virusas/?multi_permalinks=648288256333847
https://www.facebook.com/groups/1994091020875362/?multi_permalinks=3124472811170505 (closed)
https://www.facebook.com/groups/159021887526863
https://www.facebook.com/groups/412929278885515/?multi_permalinks=2097940567051036 (closed)
https://www.facebook.com/groups/3495414077220547
https://www.facebook.com/groups/lietuvos.mamyciu.turgelis
https://www.facebook.com/groups/1312138168987450
https://www.facebook.com/groups/371615077313631

Survey of participants.

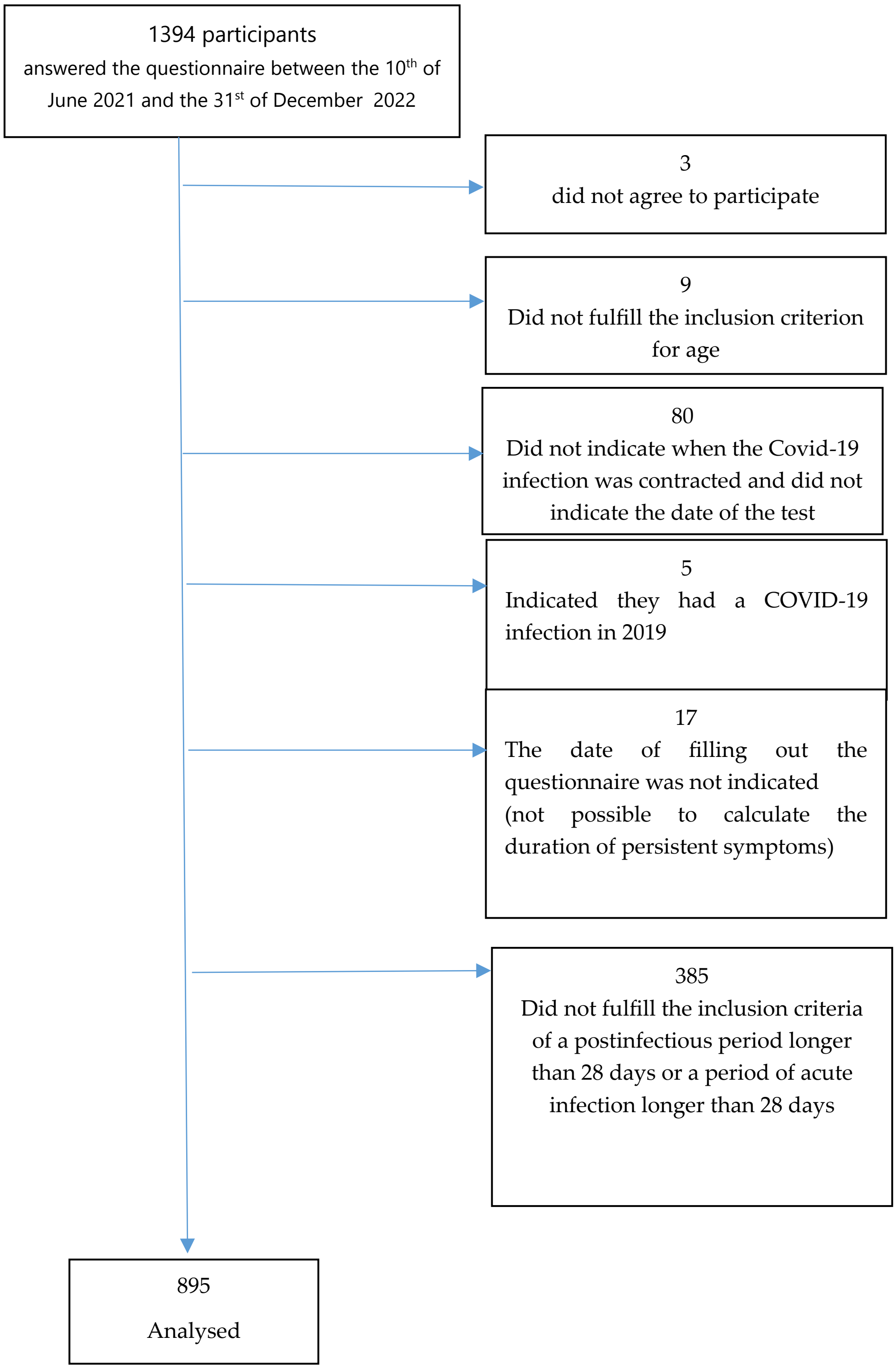

Supplemental Material S2. Persistent symptoms in 895 participants are presented as numbers and percentages in a whole cohort and among those reported as "healthy" and "unhealthy" prior the infection. The data show all persistent symptoms "healthy" vs "unhealthy" prior acute SARS-CoV-2 infection. Differences between these two latter groups are presented in p-values and p-value lower than 0.05 is indicated in a bold style.

	**All *N*=895 (%)**	**"Healthy" *N*=473 (52.8%)**	**"Unhealthy" *N*=422 (47.2%)**	**Statistics**
1. Decrease in physical capacity/absence of strength (N1)	479 (53.5%)	231 (25.8%)	248 (27.7%)	**p=0.002**
2. General fatigue (N2)	478 (53.4%)	241 (26.9%)	237 (26.5%)	p=0.068
3. Memory disorders (N3)	425 (47.5%)	207 (23.1%)	21 (24.4%)	**p=0.011**
4. Disorders of attention, concentration, concentration (N4)	356 (39.8%)	175 (19.6%)	181 (20.2%)	**p=0.042**
5. Partial/total loss of smell (Th1)	270 (30.2%)	136 (15.2%)	134 (15.0%)	p=0.183
6. Headache (Cp1)	259 (28.9%)	124 (13.9%)	135 (15.1%)	**p=0.034**
7. Palpitations/tachycardia/arrhythmia (C1)	257 (28.7%)	103 (11.5%)	154 (17.2%)	**p<0.001**
8. Hair loss (S1)	254 (28.4%)	114 (12.7%)	140 (15.6%)	**p=0.002**
9. Mood swings (sadness, confusion, irritability) (P1)	237 (26.5%)	129 (14.4%)	108 (12.1%)	p=0.311
10. Insomnia (N5)	212 (23.7%)	93 (10.4%)	119 (13.3%)	**p=0.002**
11. Head dizziness (N6)	197 (22.0%)	100 (11.2%)	97 (10.8%)	p=0.279
12. Body/muscle/bone/joint pain (Cp2)	184 (20.6%)	60 (6.7%)	124 (13.9%)	**p<0.001**
13. Various migrating/distressing pains in the body where it is difficult to pinpoint one specific painful place (Cp3)	179 (20.0%)	70 (7.8%)	109 (12.2%)	**p<0.001**
14. Anxiety (P2)	173 (19.3%)	87 (9.7%)	86 (9.6%)	p=0.252
15. Shortness of breath/labored breathing (L1)	171 (19.1%)	66 (7.4%)	105 (11.7%)	**p<0.001**
16. "Brain fog", heavy head, back pain (N7)	158 (17.7%)	81 (9.1%)	77 (8.6%)	p=0.362
17. Night sweats (N8)	154 (17.2%)	58 (6.5%)	96 (10.7%)	**p<0.001**
18. Excessive/unusual sleepiness (N9)	152 (17.0%)	74 (8.3%)	78 (8.7%)	p=0.149

19. Chest pain/pressure (C2)	148 (16.5%)	67 (7.5%)	81 (9.1%)	**p=0.027**
20. Partial/total loss of taste (Th2)	136 (15.2%)	72 (8.0%)	62 (7.2%)	p=0.528
21. Lower back pain (Cp4)	133 (14.9%)	50 (5.6%)	83 (9.3%)	**p<0.001**
22. Blurred vision (V1)	127 (14.2%)	50 (5.6%)	77 (8.6%)	**p<0.001**
23. Fluctuating symptoms (some come and go) (O1)	109 (12.2%)	44 (4.9%)	65 (7.3%)	**p=0.004**
24. Balance/equilibrium disorders (N10)	108 (12.1%)	45 (5.0%)	63 (7.0%)	**p=0.009**
25. "Dry" eyes (V2)	105 (11.7%)	42 (4.7%)	63 (7.0%)	**p=0.003**
26. Others Sleep consents (N11)	102 (11.4%)	43 (4.8%)	59 (6.6%)	**p=0.014**
27. Increased blood pressure (C3)	102 (11.4%)	27 (3.0%)	75 (8.4%)	**p<0.001**
28. Reflux/heartburn (G1)	100 (11.2%)	42 (4.7%)	58 (6.5%)	**p=0.014**
29. Dry throat/"lump" in the throat (Th3)	97 (10.8%)	40 (4.5%)	57 (6.4%)	**p=0.010**
30. Dry, flaky skin (S2)	90 (10.1%)	38 (4.2%)	52 (5.8%)	**p=0.022**
31. Hearing impairment (tinnitus/tinnitus/jamming) (N12)	87 (9.7%)	32 (3.6%)	55 (6.1%)	**p=0.001**
32. Hand numbness/cold/pain/swelling (N13)	84 (9.4%)	29 (3.2%)	55 (6.1%)	**p<0.001**
33. Blinking/blinking in the eyes (V3)	83 (9.3%)	38 (4.2%)	45 (5.1%)	p=0.108
34. Feet numbness/cold/swelling/pain (N14)	83 (9.3%)	27 (3.0%)	56 (6.3%)	**p<0.001**
35. Weight gain (E2)	82 (9.2%)	29 (3.2%)	53 (5.9%)	**p<0.001**
36. Cramps/twitches in various muscles of the body (N15)	79 (8.8%)	32 (3.6%)	47 (5.3%)	**p=0.014**
37. Dry cough (L2)	77 (8.6%)	42 (4.7%)	35 (3.9%)	p=0.424
38. Upper back/neck pain (Cp5)	74 (8.3%)	20 (2.2%)	54 (6.0%)	**p<0.001**
39. Other neuralgic pains (Cp6)	63 (7.0%)	16 (1.8%)	47 (5.3%)	**p<0.001**
40. Various skin rashes (S3)	62 (6.9%)	25 (2.8%)	37 (4.1%)	**p=0.028**
41. Congested/runny nose (Th4)	62 (6.9%)	26 (2.9%)	36 (4.0%)	**p=0.049**
42. Fear (P3)	62 (6.9%)	34 (3.8%)	28 (3.1%)	p=0.424
43. Diarrhea/constipation (G2)	61 (6.8%)	25 (2.8%)	36 (4.0%)	**p=0.037**
44. Cough/sputum production (L3)	61 (6.8%)	27 (3.0%)	34 (3.8%)	p=0.104
45. Excessive/constant thirst (E3)	57 (6.4%)	27 (3.0%)	30 (3.4%)	p=0.236
46. Lack of appetite (E4)	56 (6.3%)	25 (2.8%)	31 (3.5%)	p=0.129
47. Sore throat (Th5)	50 (5.6%)	18 (2.0%)	32 (3.6%)	**p=0.010**
48. Nausea (G3)	50 (5.6%)	25 (2.8%)	25 (2.8%)	p=0.393

49. Painful/sensitive scalp (S4)	47 (5.3%)	16 (1.8%)	31 (3.5%)	**p=0.006**
50. Weight loss (E5)	44 (4.9%)	23 (2.6%)	21 (2.3%)	p=0.529
51. Mouth sores/ulcers (Th6)	34 (3.8%)	13 (1.5%)	21 (2.3%)	p=0.059
52. Kidney pains/urination problems (O2)	32 (3.6%)	11 (1.2%)	21 (2.3%)	**p=0.025**
53. Decreased blood pressure (C4)	31 (3.5%)	16 (1.8%)	15 (1.7%)	p=0.516
54. Prolonged elevated body temperature (E6)	31 (3.5%)	17 (1.9%)	14 (1.6%)	p=0.484
55. Pain in the nasal area (Th7)	30 (3.4%)	11 (1.2%)	19 (2.1%)	p=0.052
56. Anaemia (O3)	28 (3.1%)	12 (1.3%)	16 (1.8%)	p=0.188
57. Chills without fever (E7)	27 (3.0%)	13 (1.5%)	14 (1.6%)	p=0.381
58. Abnormally low body temperature (E8)	21 (2.3%)	10 (1.1%)	11 (1.2%)	p=0.395
59. Changed salivation (Th8)	20 (2.2%)	7 (0.8%)	13 (1.5%)	p=0.082
60. Other symptoms (record if you didn't find the right one) (O4)	19 (2.1%)	8 (0.9%)	11 (1.2%)	p=0.237
61. Fever/ chills (E9)	18 (2.0%)	6 (0.7%)	12 (1.3%)	p=0.075
62. Inflammations/infections of other body organs/body systems (O5)	18 (2.0%)	6 (0.7%)	12 (1.3%)	p=0.075
63. Eye inflammation/ "barley" (V4)	14 (1.6%)	6 (0.7%)	8 (0.9%)	p=0.313
64. Vomiting (G4)	4 (0.4%)	1 (0.1%)	3 (0.3%)	p=0.271

Abbreviations for remaining symptoms related to: N – nervous system (15 symptoms); Ch-chronic pain (6 symptoms); Th – throat, nose and ear (8 symptoms); C – cardiovascular (4 symptoms); P – psychological (3 symptoms); S – skin (4 symptoms); L – lung (3 symptoms); E- endocrine (9 symptoms); G- gastrointestinal (3 symptoms); V – vision and eyes (4 symptoms); O- others (5 symptoms).

Supplemental Material S3. Principles of grouping comorbidities.

HEART DISEASES:<br>• Arterial hypertension/hypotension<br>• Arrhythmia<br>• Tachycardia<br>• Heart failure<br>• Pericarditis	ENDOCRINE DISEASES:<br>• Diabetes mellitus<br>• Hypothyroidism/ Hyperthyroidism<br>• Obesity<br>• Other metabolic diseases	PSYCHIATRIC DISEASES:<br>• Anxiety<br>• Depression<br>• Other/various psychiatric disorders
DISEASES OF THE NERVOUS SYSTEM:<br>• Sleep disorders<br>• Migraine<br>• Headache<br>• Chronic pain syndrome<br>• Various neuralgias, radiculopathy	DISEASES OF THE DIGESTIVE SYSTEM:<br>• Inflammation of the digestive tract<br>• Liver function disorders<br>• Reflux<br>• Ulcers	SKIN DISEASES:<br>• Allergies<br>• Rashes<br>• Psoriasis<br>• Dry, sensitive skin
RHEUMATIC/INFLAMMATORY DISEASES:<br>• Various arthritis<br>• Inflammation of the joints<br>• Bone/joint/muscle pain	RESPIRATORY SYSTEM DISEASES:<br>• Pneumonia<br>• Bronchitis<br>• Asthma<br>• Other lung diseases	KIDNEY DISEASES:<br>• Kidney failure<br>• Inflammations
	OTHER:<br>• Anemia<br>• Visual disturbances<br>• Lack of vitamins/micronutrients<br>• Coagulation disorders	

	• Various unspecified inflammations/diseases	

Supplemental Material S4. Principles of grouping medication.

Drugs acting on the cardiovascular system	• Anti-arrhythmic<br>• Anti-hypertensive<br>• Anti-hypotensive<br>• Regulating blood circulation:<br>• anti-anemics<br>• statins<br>• anti-thrombotic
Supplements/vitamins	• Vitamins<br>• Supplements<br>• Micro elements
Anti-asthmatic drugs	• Anti-asthmatics<br>• Inhalers
Nonsteroidal anti-inflammatory drugs and antibiotics	• NSAIDs<br>• Antibiotics
Drugs acting on the endocrine system	• Various hormones<br>• Glucocorticoids<br>• Anti-diabetics
Psychopharmacological drugs	• Psycholeptics<br>• Psychoanaleptics<br>• Antidepressants
Drugs acting on the gastrointestinal tract	• Anti-reflux
Other	• Anti-tumor<br>• Anti-allergic<br>• Homeopathic<br>• Other unspecified

Names of drugs as indicated in the survey.

**DRUGS ACTING ON THE CARDIOVASCULAR SYSTEM**	
Anti-hypertensive	• Agen (*amlodipine*)<br>• Amlodipine<br>• Betalok (*metaprolol*)<br>• Cosimprel (*perindopril and bisoprolol*)<br>• Kaptopril<br>• Lisinopril<br>• Metoprololum (*metaprolol)*<br>• Nebilet (*nebivolol)*<br>• Nebivololum<br>• Noliprelis (*perindopril and indapamide*)<br>• Tarka (*trandolapril and verapamil*)<br>• Ramiprilum<br>• Valsakombi (*valsartan and hydrochlortiazide)*<br>• Valsartanum<br>• Viacorma (*perindopril and amlodipine)*<br>• Zovanat (*meldonium*)<br>• Trental (*pentoxifylline*)<br>• Preductal (*trimetazidine*)

	• Mildronat (*meldonium*)<br>• Ivabradin<br>• Implicor (*metaprolol and ivabradine)*<br>• Others: antihypertensive
Anti-arrhythmics	• Rhytmonorm (*propafenone*)<br>• Others: drugs regulating heart pulse
Anticoagulants	• Apixabanum<br>• Aspirine (*acetylsalicylic acid*)<br>• Eliquim (*apixabanum*)<br>• Trombo (*acetylsalicylic acid*)<br>• Xarelto (*rivaroxabanum*)<br>• Anti-thrombosis (*acetylsalicylic acid*)
Anti-anaemics	• Tardiferon
Statins	• Atoris (*atorvastatinum*)
Anti-vertigo	• Betahistine<br>• Betaserc (*betahistine*)<br>• Reducing dizziness
**SUPPLEMENTS/ VITAMINS**	
Vitamins	• A, B, C, D, E,<br>• Vit. Oxylyc<br>• Vit. against hair loss<br>• Vit. for energy<br>• Vit. To strengthen immunity<br>• Milgamma (*benfotiamine/piridoksin/cyanocobalamin)*<br>• Multivitamins<br>• Minerals<br>• Neurorubine *(tiamine/piridoksin/ cyanocobalamin)*<br>• Well women (*multivitamin for women*)<br>• Vitamins<br>• Others: neuro-injection ampulle
Supplements and OTC drugs	• Antioxidants<br>• Arthryl (*glucosamine sulfate*)<br>• Aterolip (*red yeast fermented rice powder)*<br>• Astaksantin<br>• Atlant (*collagen/ vitamin C, calcium)*<br>• Atmosil (*food supplement with herbal extracts, pterostilbene, quercetin, lycopene and algae*)<br>• Beauty Collagen<br>• Biozink<br>• Cantalin (*diosmin/hesperidin*)<br>• Cardiopill (*vitamin C/ CoQ 10*)<br>• Carsil (*silimarin*)<br>• Cerebrum Compositum (*cerebrum suis, embryo suis, hepar suis, placenta suis, kalium phosphoricum, selenium, thuja, ignatia, bothrops lanceolatus, acidum phosphoricum, china, manganum phosphoricum, magnesium phosphoricum, anacardium, conium, luesinum-*

	nosode, medorrhinum-nosode, hyoscyamus, aconitum, cocculus, ambra, sulfur, kalium bichromicum, gelsemium, ruta, arnica, aesculus)<br>• Chlorofilum<br>• Cholesterol Balance (*hop cone,*<br>• *plantain seeds, red yeast, monacolin K, burdock root, berberine hydrochloride,*<br>*vegetable artichoke leaf, choline, policosanols)*<br>• Chromas<br>• Cinie<br>• Zinc<br>• Cordycepts<br>• Essentiale Forte (*phospholipida ex soia*)<br>• Eukamirsan (*eucalyptus, myrtle, orange and lemon essential oils*)<br>• Echinacae preparations<br>• Felxit (*calcium, vitamin C, magnesium, vitamin B6, Vitamin D3, chondroitin, glucosamine sulfate, collagen, hialuronic acid, proline)*<br>• Feroglobin/Ferrotab<br>• Flavamed (*ambroxol*)<br>• Folic acid<br>• Iron supplements<br>• Gelomyrtol *(eucalyptus, sweet orange, myrtle, lemon essential oils)*<br>• Probiotics<br>• Preparations of Gink<br>• Hawley Extract<br>• Hepamax (*silimarin/vitamin C*)<br>• Imuneco (*ginseng, CoQ 10*)<br>• Immune boosting supplements<br>• Imunosil Virufix (*cink, vitamin C*)<br>• Calcium<br>• Potassium<br>• Kardicept (*essential oils, antioxidants*)<br>• Koferm Q10<br>• Kvercetin<br>• Lepicol (*plantain peels, inulin, lactic ascid bacteria*)<br>• Magnesium<br>• Miscellanous supplements<br>• Melatonin<br>• Memory Plus (*phosphatidylserine/choline*)<br>• Supplements to improve sleep<br>• Evening Primrose oil<br>• Nataspin (*nattokinase, monacolin, policonasolum, polyphenol*)<br>• Neurozan (*ginseng, fostatidilcholin, fostatidilserin, CoQ 10)*<br>• Omega-3<br>• Panangin (*potassium, magnesium*)<br>• Sedatif PC (b*elladonna, calendula officinalis, chelidonium majus, abrus precatorius, viburnum opulus, aconitum napellus)*<br>• Selenus<br>• Spirulina (*spirulina platensis*)

	• Stop Stress (*valerian, passionflower*)<br>• Stress Plex (vitamin C, zinc)<br>• Valerosan (*valerian root extract, lemon balm leaft powder, fenugreek grass powder*)<br>• Fishoil<br>• Others: additional insomnia relief
**DRUGS ACTING ON THE IMMUNE SYSTEM (ANTI-ASTHMATIC and ANTI-ALLERGIC)**	
Anti-asthmatics	• Easy Haler (*budesonidum*)<br>• Inhaler (*salbutamolum*)<br>• Interferon beta<br>• Montelucast (*montelucastum*)<br>• Salbutamolum<br>• Others: against asthma
Anti-allergic	• Bilastinum<br>• Cirrus (*cetirizinum*)<br>• Fexofenadine<br>• Tavegil (*clemastinum*)<br>• Others: anti-allergic
**NON-STEROID ANTI-INFLAMMATORY and ANTI-BACTERIAL DRUGS**	
Antibiotics	• Amoxicillin<br>• Amoxiklav (*amoxicillinum and acidum clavulanicum*)<br>• Ospamox (*amoxicillinum*)<br>• Others: antibiotics
NSAID (Non-steroidal anti-inflammatory drugs):	• Appap (*paracetamol*)<br>• Arcoxia (*etoricoxib*)<br>• Brexin (*piroxicam*)<br>• Citramon (*acetylsalicylic acid, paracetamol, caffeine)*<br>• Diclovit (*diclofenac and vitamine* B_1, B_6, B_{12})<br>• Fremanezubam<br>• Ibumetin (*ibuprofen)*<br>• Ibuprofen<br>• Migard (*frovatriptan*)<br>• Movali (*meloxicam*)<br>• Naproxen<br>• Nimesil (*nimesulid*)<br>• Plascledine (*avocado and soyabean oil*)<br>• Scudex (*tramadol/dexketoprofen*)<br>• Vimovo (*naproxenum/esomeprazole*)<br>• Others: oinments for pain, against pain, againt inflammation
**DRUGS ACTING ON THE ENDOCRINE SYSTEM**	
Hormones	• Hormones<br>• L-Thyroxine (*levothyroxine*)<br>• Thyroxine
Glucocorticoids	• Medrol (*methylprednisolone*)<br>• Prednisolon
Anti-diabetes	• Metformin (*metformin*)<br>• Tivulin (*metformin*)

PSYCHOPHARMACOLOGICAL DRUGS	
Antidepressants	• Amytryptilin<br>• Cipralex (*escitalopram*)<br>• Escitalopram<br>• Mirtazepine (*mirtazapin*)<br>• Antidepressants
Psycholeptics	• Zolpidem<br>• Zopiclone<br>• Xanax (*alprazolam*)<br>• Quetiapine<br>• Lexotanil (*bromazepam*)<br>• Halcion (*triazolamum*)<br>• Bromazepamum (*bromazepam*)
Psychoanalyptic:	• Cavinton (*vinpocetinum*)<br>• Noritren (*nortriptyline*)<br>• Pyracetamum (*piracetam*)<br>• Pramistar (*pramiracetam*)<br>• Tanakan (*ginkgo folium)*<br>• Valdaxan (*agomelatine*)
**DRUGS ACTING ON THE GASTROINTESTINAL SYSTEM**	
Anti-reflux	• Malox (magnesium hydroxide/aluminium hydroxide)<br>• Nexium (*esomeprazole*)<br>• Omeprazol<br>• Others: against reflux
**OTHER DRUGS**	
Other drugs	• ACC600 (*acetylcisteine*)<br>• Ambio<br>• Ambroxol<br>• Relaxing nights<br>• Against vomiting<br>• Biological therapy<br>• Bloxivir (*oral spray gel*)<br>• Thyme Tea<br>• Detralex (*diosminum/hesperidinum*)<br>• Enterol (*Saccharomyces boulardii)*<br>• Floconazole<br>• Hydroxizyne<br>• Buckwheat Tea<br>• Other<br>• Creams for the treatment of skin disease<br>• Eye drops<br>• Mysimba (*bupropion/naltrexone*)<br>• Nasogast<br>• Nasonex (*mometasone furoatum)*<br>• Pangrol (*pancreatinum*)<br>• Plaquenil (*hydroxychloroquinum*)<br>• Rehydron (*powder for oral solution glucose, sodium chloride, sodium citrate and potassium chloride*)<br>• Roswera (*rosuvastatin*)<br>• Sinupret (*verbena herb, gentian root, sorrel herb, elderflower, primrose flower)*<br>• Smecta (*diosmectite*)

	• Strepsils (*flurbiprofen)*<br>• Improve brain functions<br>• Non-prescribed sedative medicines<br>• Expanding drugs<br>• Heart dropps<br>• Medicinal herbs<br>• Valerian<br>• Herbal Teas/Tinctures<br>• Olynth (*xylometazolin*e)<br>• Others: anti-anxiety, anti-insomnia, anti-cough, anti-hair loss.
